# mtTB: A Web-Based R/Shiny App for Pulmonary Tuberculosis Screening

**DOI:** 10.3389/fcimb.2022.850279

**Published:** 2022-03-18

**Authors:** Zhougui Ling, Shuangping Huang, Zhongwei Wen, Zhenming Tang, Ying Huang, Ni Wei, Mei Liu, Jinyan Wu

**Affiliations:** Department of Pulmonary and Critical Care Medicine, The Fourth Affiliated Hospital of Guangxi Medical University, Liuzhou, China

**Keywords:** TB, peripheral blood, mitochondria-derived small RNAs, ratio-based method, biomarkers

## Abstract

Pulmonary tuberculosis caused by Mycobacterium tuberculosis remains a global issue. However, the diagnosis of active pulmonary tuberculosis (TB) remains a challenge in the clinic. Small non-coding RNAs are potential diagnostic biomarkers for pulmonary tuberculosis. However, the current normalization methods are not stable and usually fail to reliably detect differentially expressed sncRNAs. To identify reliable biomarkers for pulmonary tuberculosis screening, we utilized the ratio-based method on the newly discovered mitochondria-derived small RNAs in human peripheral blood mononuclear cells. The prediction model of seven mtRNA biomarkers noteworthily enables the discrimination between pulmonary tuberculosis patients and controls in discovery (AUC = 0.906, 23 patients) and independent validation cohort (AUC = 0.968, 20 patients). Moreover, we present mtTB (https://tuberculosis.shinyapps.io/mtTB/), a novel R Graphical User Interface (GUI) that provides reliable biomarkers for the feasibility of blood-based screening, and produce a more accurate tool for pulmonary tuberculosis diagnosis in real clinical practice.

## Introduction

Pulmonary tuberculosis (TB) is a chronic pulmonary infectious disease caused by Mycobacterium tuberculosis (Mtb) and is the second most predominant infectious disease across the world (Bando-Campos et al., 2019). Current diagnostic tools comprise smear microscopy, microbiological culture, and molecular detection by Xpert MTB/RIF (Xpert) or Xpert MTB/RIF Ultra (Ultra). However, there are additional shortcomings of each approach, such as the insufficient sensitivity of microscopy, the time delay for culture, the high cost of molecular tests, and false-positive Ultra results (Turner et al., 2020).

Mitochondria are critical organelles for maintaining cell energy metabolism and play an important role in the development and progression of lung cancer (Roberts and Thomas, 2013). The human mitochondrial DNA (mtDNA) encodes 37 genes including 2 rRNAs, 22 tRNAs, and 13 protein-coding genes (Larriba et al., 2018). Moreover, approximately 12% of the unique small RNAs identified were encoded in the mitochondrial genome (Riggs and Podrabsky, 2017; Hirose et al., 2019). Recent studies reveal different types of sncRNAs that are associated with the mitochondrial genome, and these sncRNAs generated from the mitochondrial DNA were proposed to regulate and communicate with various pathways that interact with the nuclear genome (Larriba et al., 2018). Therefore, mitochondrial-derived RNAs (mtRNAs) could play an important role in pathophysiological processes and infectious diseases. Since various sncRNAs, such as miRNA, snoRNA, and piRNA, are widely studied in the diagnosis of TB, no clear research has been given for mitochondria-derived small RNAs (mtRNAs) (Wang et al., 2011; de Araujo et al., 2019). Moreover, most studies of sncRNA normalization methods are based on synthetic external spiked-in controls or published endogenous miRNA controls. However, those references are too labile to use directly in sncRNA studies. The ratio-based method provides a solution for the difficult normalization problem for sncRNA data to identify reliable biomarkers to reach the real clinical application (Deng et al., 2019). Here, we aim to develop a new reliable model to predict TB patients based on peripheral blood mtRNAs and the ratio-based method.

## Methods

### Datasets

Discovery and the independent validation set were downloaded from the Gene Expression Omnibus (GEO) repository (GSE148861, GSE148862). Each small RNA-seq was aligned using SPORTS1.1 software to extract mtRNA expression levels (Shi et al., 2018). At first, all miRNA-seq FASTQ files removed adapter sequences from raw reads using nf-core/smrnaseq software (Ewels et al., 2020). The trimmed sequence reads were aligned to the mitotRNAdb database using the STAR algorithm (Jühling et al., 2009). Raw counts from mapped reads were obtained using the htseq-count script from the HTSeq tools (Anders et al., 2015). Missing values were imputed by MetImp 1.2 (Wei et al., 2018).

### Ratio-Based Normalization Method and Shiny App

To stabilize the mtRNA expression profile, we performed the ratio-based method to the mtRNAs (Deng et al., 2019). The mtRNA paired ratios were calculated according to the equation: Ratio(mtRNA1_to_mtRNA2) =mtRNA1/mtRNA2. All results are displayed as the mean ± SEM. Differentially expressed (DE) mtRNA analysis was performed on the discovery group derived from GSE148861 using unpaired Student’s t-tests. mtTB uses Shiny’s reactivity with built-in R functions from packages for prediction model analysis and best subset selection including “survminer”, “shiny”, “precrec”, “glmnet”, and “randomForest”. Statistical significance was assigned as p < 0.05.

### Prediction Model Construction

**Figure 1:**
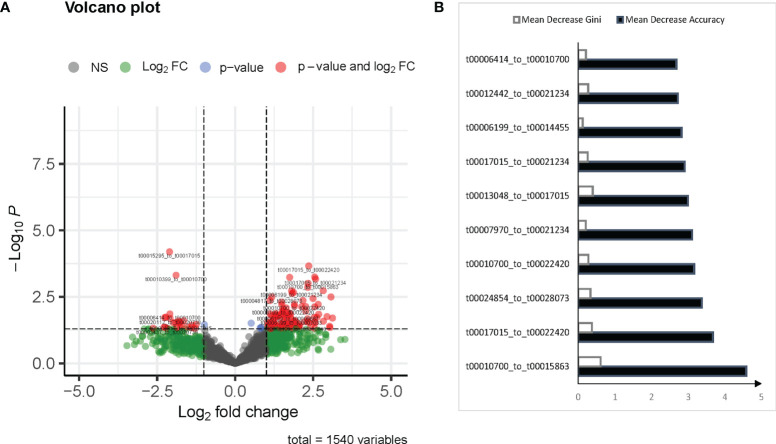
mtRNA diagnostic panel. **(A)** Volcano plot of differentially expressed mtRNAs between normal and pulmonary tuberculosis (TB). **(B)** Mean Decrease Accuracy shows the relative degree to which a factor improves the accuracy of the forest in classification prediction; Mean Decrease Gini assigns a weight of importance to each parameter, which improves accuracy of the prediction.

Differentially expressed (DE) (p value < 0.01) and | Log2(fold change) | > 1 paired mtRNAs were enrolled using the randomForest prediction model. The Mean decrease of accuracy and mean decrease Gini of each paired mtRNA were calculated by the randomForest model. Feature selection based on the overlap of the top 10 Mean decrease of accuracy and mean decrease Gini (**Figure 1B**). The final prediction model was built by selected features.

## Results

### Patient Cohorts and the Molecular Signature Composed of mtRNAs

The clinical characteristics of the two TB cohorts are summarized in **Supplementary Table 1**. Our study included 43 cases, composed of 18 patients with TB and 25 healthy controls. In the discovery cohort, the average age of the TB group was 35 ± 1.1, and the control group was 52.7 ± 1.0. In the validation dataset, the average age of the TB group was 38.1 ± 2.8, and the control group was 45 ± 1.85. In total, we identified 9 mtRNA species in human peripheral blood samples (**Supplementary Table 2**). There are 7 types of mtRNAs by grouping mtRNA species into subcategories according to their parent tRNA types (i.e., mt-tRNA-GAA, mt-tRNA-Ser-GCT_5_end). The mtRNA sequences length ranged from 15 to 32 nt with an average length of 19.6 nt.

### Dysregulated mtRNAs in TB and Prediction Model Construction

**Figure 2:**
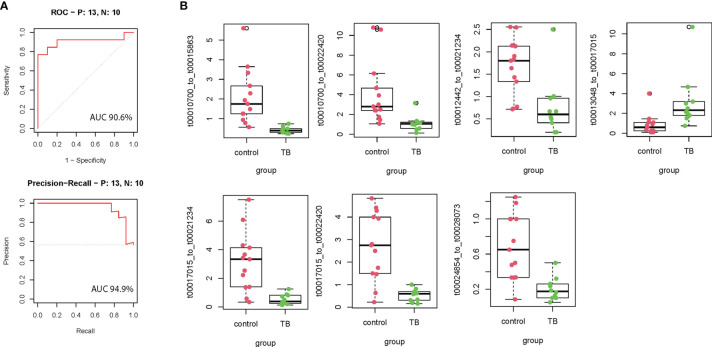
The performance of the training model in the discovery cohort. **(A)** Seven model selected mtRNA expression levels in the discovery cohort. **(B)** ROC curve and PR curve of the diagnostic prediction model with mtRNA markers in the discovery cohort; All box plots are statistical significant, p <0.01.

According to the inclusion criteria described in *Methods*, 127 mtRNA pairs were significantly different by Student t-test (**Figure 1A** and **Supplementary Table S3**). The random forest (RF) algorithm was performed to select the most effective variables from 127 mtRNA pairs to construct prediction models. According to the RF mean decrease of accuracy and RF mean decrease GINI (**Figure 1B**), seven mtRNA pairs were selected, including upregulated t00013048_to_t00017015 and downregulated t00010700_to_t00015863, t00010700_to_t00022420, t00012442_to_t00021234, t00017015_to_t00021234, t00017015_to_t00022420, and t00024854_to_t00028073 in TB samples (**Figure 2B**). Out-of-bag (OOB) estimations were used to assess the predicted error. We evaluated the model performance by a receiver operating characteristic curve (ROC curve) and Precision–Recall curve (PR curve). The area under the receiver operating characteristic (ROC) curve (AUC) was 0.906 between TB and control subjects and 0.949 (AUC) in the PR curve (**Figure 2A**).

### The Prediction Model in the Independent Validation Cohort and mtTB

**Figure 3:**
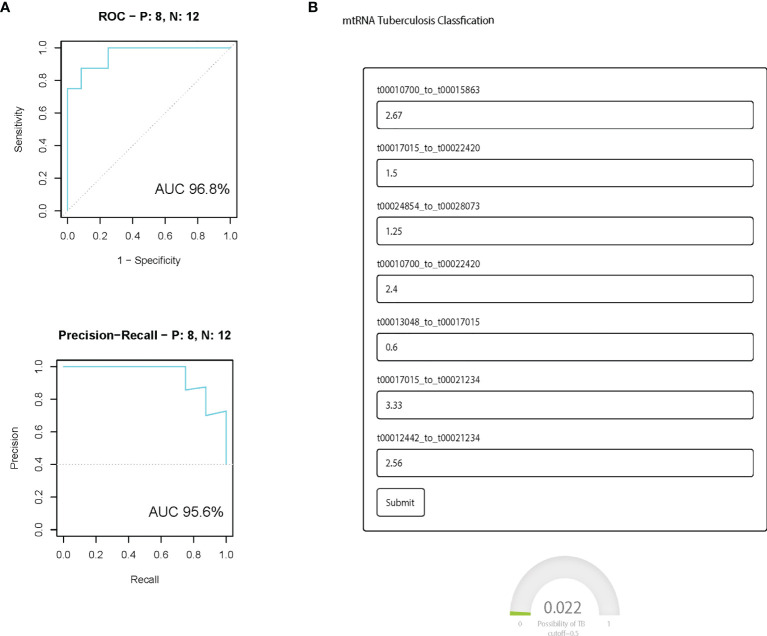
The performance of the prediction model in the independent validation cohort. **(A)** ROC curve and PR curve of the diagnostic prediction model with mtRNA markers in the independent validation data set; All box plots are statistical significant, p <0.01. **(B)** Screenshot of the mtTB diagnosis tool.

We further evaluated the prediction model in the independent validation cohort. The boxplot shows eight selected mtRNA expression levels in the validation dataset (**Figure 3A**). There is significant variation remaining, except for the t00016493_to_t00024522, which is marginal for the validation cohort. For the prediction model, the AUC was 0.968 between TB and non-TB cases and 95.6 (AUC) in the PR curve which infers the strong classification power for TB screening. At the same time, we have developed a user-friendly webpage where doctors only need to input the mtRNA pairs to get the probability of TB diagnosis (**Figure 3B**).

## Discussion

Globally, M. tuberculosis drastically affects not only TB patients but also asymptomatic undiagnosed subjects in the community. Fast and precise diagnosis is critical for the control of TB spread and sufficient antimicrobial therapy. Although there are multiple methods in the clinical diagnosis of pulmonary TB, such as sputum smear which provides rapid results and is widely used in clinical laboratories, this traditional method shows a low positive rate of 20% to 30%. Moreover, the gold standard of pulmonary TB diagnosis requires a long incubation time (4–8 weeks) (McNerney et al., 2012). In the early stage of TB infection, one unmet challenge in TB diagnosis is to accurately differentiate other lung diseases from TB with similar clinical symptoms and radiological features.

Circulating small non-coding RNAs have been broadly explored as novel and non-invasive diagnostic and prognostic biomarkers. Many studies have shown that circulating miRNAs serve as potential biomarkers for the detection of TB. However, the performance of miRNA-based TB diagnostic signatures is limited (Zhang et al., 2013; Latorre et al., 2015). Interestingly, our mtRNA signature, derived from the PBMC non-canonical sncRNAs, shows superiority over the miRNA-based signature in diagnosing pulmonary tuberculosis (Pedersen et al., 2019). Compared with miRNA, non-canonical small RNAs such as tsRNAs exhibit a surprising complexity and variability in their sequence (Shi et al., 2019). Moreover, their extraordinary performance in cancer diagnosis and prognosis may be due to the additional complex coating of non-canonical small non-coding RNAs (Gu et al., 2020; Zhu et al., 2021; Zuo et al., 2021).

An affordable, reproducible, and non-invasive method for predicting the severity of TB is required to support longitudinal management and clinical decision-making. In this study, we aimed to develop blood-based screening to improve the sensitivity and specificity of classifications between normal and TB patients. To our knowledge, this is the first time that machine learning algorithms have been used to diagnose TB by mtRNA on the clinical system. Furthermore, this algorithm has been implemented into a user-friendly Shiny app, an R package that makes it easy to build interactive web apps straight from R, to support further independent investigations of its clinical practice (Sievert, 2020). Previous miRNA-based TB diagnostic tools were either inaccurate or difficult to use (Zhou et al., 2016; Sampath et al., 2021). This shiny app only needs to input the expression ratio of mtRNA, and the result can be obtained quickly after clicking submit, which greatly reduces the user’s time.

However, the model was established for the diagnosis of TB and the questions that require further investigation still remain. First, the mtRNA-based signature functions are unknown. Second, large-scale, multicenter case–control studies are warranted to validate our results and identify the signature. Third, since we obtained the sequences of mtRNA signature, all candidate genes need to be validated by quantitative PCR.

## Data Availability Statement

The datasets presented in this study can be found in online repositories. The names of the repository/repositories and accession number(s) can be found in the article/**Supplementary Material**.

## Ethics Statement

The Ethics Committee of Bengbu Medical College approved this study, with written informed consent obtained from all subjects, which conformed to the standard indicated by the Declaration of Helsinki. The patients/participants provided their written informed consent to participate in this study.

## Author Contributions

ZL and SH had the design and launched the study. ZL and SH processed the statistical data analyses, and all authors revised the manuscript and approved the version for publication. All authors contributed to the article and approved the submitted version.

## Funding

This work was supported in part by funding from the Key Research and Development Program of Guangxi Zhuang Autonomous Region (No. AB16380152), in part from the Key Research and Development Program of Liuzhou (2018BJ10509) and in part from the ‘139’ Incubation Program for high-level medical talents in Guangxi.

## Conflict of Interest

The authors declare that the research was conducted in the absence of any commercial or financial relationships that could be construed as a potential conflict of interest.

## Publisher’s Note

All claims expressed in this article are solely those of the authors and do not necessarily represent those of their affiliated organizations, or those of the publisher, the editors and the reviewers. Any product that may be evaluated in this article, or claim that may be made by its manufacturer, is not guaranteed or endorsed by the publisher.
