# Supplementary material for: mtTB: A Web-Based R/Shiny App for Pulmonary Tuberculosis Screening
Source: Front Cell Infect Microbiol. 2022 Mar 18;12:850279. doi: 10.3389/fcimb.2022.850279 (PMC8982078; doi:10.3389/fcimb.2022.850279)
Supplement: Supplementary file 1 [file DataSheet_1.pdf]

mtTB: a web-based R/shiny app for pulmonary tuberculosis screening

Supplementary Table 1. Clinical characteristics of two tuberculosis cohorts

**Cohort**	**Discovery**	**Validation**
**Num. of patients**	23	20
**Age in years, mean (SD)**		
Control	52.7(1.0)	45(1.85)
Tuberculosis	35(1.1)	38.1(2.8)
**Males, count (%)**		
Control	7/13(53.8)	6/12(50.0)
Tuberculosis	7/10(70)	5/8(62.5)

Supplementary Table 2. The mitochondria-derived RNA (mtRNA) signature

names	Sequences	Length
t00010700	ACTTTTTCTCTGACCA	16
t00017015	CTTTTTCTCTGACCA	15
t00024854	CACTGTAAAGCTAACTTAGCATTA	24
t00013048	TAGGATGGGGTGTGATAGG	19
t00012442	GGTAAAATGGCTGAGTGAAGCATTGGACTGTA	32
t00015863	GTTTAGACGGGCTCACA	17
t00022420	TTTCCAAGGACACCA	15
t00028073	GAGAAAGCTCACAAGAACTGCTA	23
t00021234	TTTTCCAAGGACACCA	16

Supplementary Table 3. A list of mtRNAs that p-values less than 0.05 when the unpaired student t-test was evaluated

names	pvalue	Fold_change	log_FC	FDR
t00017015_to_t00027940	0.019828154	8.698174684	3.120712683	0.56259193
t00017015_to_t00022458	0.0031863	8.397414571	3.069945214	0.352911303
t00010700_to_t00015295	0.045115517	8.192511637	3.034305817	0.568363584
t00017015_to_t00027084	0.040165206	8.15445665	3.02758875	0.568363584
t00006199_to_t00022458	0.017473241	7.701777916	2.945191523	0.554823009
t00007970_to_t00022458	0.026217779	7.091588681	2.826108861	0.568363584
t00010700_to_t00022458	0.001861963	7.072595381	2.822239728	0.286742244
t00017015_to_t00022385	0.014281025	6.397756788	2.677566149	0.554823009
t00017015_to_t00028073	0.006089253	6.320137718	2.659955996	0.456715016
t00017015_to_t00020112	0.018680851	6.143608739	2.61908634	0.554823009
t00017015_to_t00021234	0.000676168	5.964851664	2.576486261	0.173549732
t00007970_to_t00014455	0.029557311	5.921470668	2.565955531	0.568363584
t00006199_to_t00014005	0.048475335	5.871031697	2.553614046	0.568363584
t00010700_to_t00014005	0.007556853	5.856186329	2.549961457	0.465502163
t00017015_to_t00024059	0.000568728	5.853501315	2.549299842	0.173549732
t00007970_to_t00014005	0.028154227	5.845095687	2.547226642	0.568363584
t00006199_to_t00014455	0.018436861	5.805714535	2.537473637	0.554823009
t00017015_to_t00024222	0.003640816	5.640710044	2.495876778	0.373790418
t00007970_to_t00028073	0.04388599	5.350813136	2.419758147	0.568363584
t00006199_to_t00028073	0.029282728	5.174407013	2.371393539	0.568363584
t00017015_to_t00022420	0.000217317	5.123415546	2.35710591	0.167334077
t00010700_to_t00015863	0.000976412	5.044584003	2.334735304	0.214810599
t00010700_to_t00013099	0.026463655	5.016855981	2.326783523	0.568363584
t00010700_to_t00014455	0.001388688	5.01524599	2.326320464	0.267322351
t00017015_to_t00027162	0.021100326	5.003638524	2.322977569	0.56259193

t00010700_to_t00027940	0.010773598	4.788589305	2.259600708	0.554823009
t00010700_to_t00028073	0.007283616	4.78137465	2.257425454	0.465502163
t00010700_to_t00029490	0.027200848	4.650162034	2.217280987	0.568363584
t00017015_to_t00029490	0.004345642	4.509771706	2.173054403	0.395153564
t00010700_to_t00021234	0.00738478	4.376376353	2.12973681	0.465502163
t00007970_to_t00010399	0.039246559	4.18977224	2.06687182	0.568363584
t00007970_to_t00013048	0.032615601	4.127378956	2.045225906	0.568363584
t00010700_to_t00026687	0.040584108	4.109884503	2.039097852	0.568363584
t00011350_to_t00015295	0.043644816	4.08529643	2.030440764	0.568363584
t00007970_to_t00015863	0.045559495	3.973256637	1.990321982	0.568363584
t00006199_to_t00015863	0.039071643	3.948439629	1.981282632	0.568363584
t00006199_to_t00010399	0.027076571	3.8587788	1.948144345	0.568363584
t00006199_to_t00013048	0.036151727	3.838373885	1.940495247	0.568363584
t00009731_to_t00015295	0.046553485	3.77249531	1.915519107	0.568363584
t00017015_to_t00026874	0.024743626	3.748701398	1.906390913	0.568363584
t00010700_to_t00022420	0.005977228	3.724898815	1.897201236	0.456715016
t00010700_to_t00020112	0.007046799	3.724176793	1.896921562	0.465502163
t00012442_to_t00028073	0.002152636	3.655825005	1.870197014	0.301369072
t00007907_to_t00022458	0.026433345	3.634345794	1.861695693	0.568363584
t00007970_to_t00021234	0.002370796	3.54992164	1.827787179	0.304252208
t00006199_to_t00021234	0.001861778	3.504548792	1.80922871	0.286742244
t00006199_to_t00007033	0.021493674	3.496451607	1.805891538	0.56259193
t00010700_to_t00016527	0.045627673	3.476695003	1.797716511	0.568363584
t00010700_to_t00027162	0.014920757	3.4097354	1.769659789	0.554823009
t00024854_to_t00027940	0.013019696	3.400673121	1.765820338	0.554823009
t00010700_to_t00026874	0.041436806	3.363898215	1.750134053	0.568363584
t00024854_to_t00028073	0.000585043	3.355708448	1.746617376	0.173549732
t00007970_to_t00024222	0.033089292	3.345669421	1.742294902	0.568363584

t00006199_to_t00009667	0.014940847	3.239022896	1.695558666	0.554823009
t00024854_to_t00030441	0.029501504	3.217956784	1.686144952	0.568363584
t00007907_to_t00014005	0.016838091	3.192303271	1.674597715	0.554823009
t00007970_to_t00009667	0.012374964	3.189738619	1.673438208	0.554823009
t00007970_to_t00024059	0.032782159	3.134575265	1.648269971	0.568363584
t00010700_to_t00024059	0.018780887	3.123247089	1.64304671	0.554823009
t00009731_to_t00022458	0.008305503	3.113899295	1.638722288	0.491941324
t00006199_to_t00024222	0.03953429	3.052356409	1.609923429	0.568363584
t00016493_to_t00028073	0.048126598	2.978552175	1.574611231	0.568363584
t00007970_to_t00016527	0.027649571	2.928734745	1.550277535	0.568363584
t00006199_to_t00022420	0.008732838	2.914359155	1.543178681	0.4980952
t00007970_to_t00022420	0.01081026	2.888528247	1.530334603	0.554823009
t00012442_to_t00024059	0.01454939	2.885387479	1.528765071	0.554823009
t00015616_to_t00024059	0.011015637	2.873303687	1.522710483	0.554823009
t00017015_to_t00024522	0.012173914	2.871626919	1.521868327	0.554823009
t00007970_to_t00015882	0.035469957	2.853263722	1.512613099	0.568363584
t00007907_to_t00014455	0.021553847	2.839396626	1.505584388	0.56259193
t00012442_to_t00027940	0.006227932	2.820153218	1.495773546	0.456715016
t00024854_to_t00027084	0.004934476	2.8031217	1.487034382	0.422171871
t00009731_to_t00027940	0.043653671	2.775294378	1.472640807	0.568363584
t00020738_to_t00024059	0.048564308	2.754171593	1.461618447	0.568363584
t00006199_to_t00024059	0.047671071	2.751323958	1.460126022	0.568363584
t00006199_to_t00016527	0.032946667	2.724576589	1.446032046	0.568363584
t00015616_to_t00024222	0.042381086	2.713462532	1.440134988	0.568363584
t00011350_to_t00024059	0.01534036	2.710129616	1.438361852	0.554823009
t00007970_to_t00027162	0.016284069	2.550403424	1.350725471	0.554823009
t00011350_to_t00028073	0.016676517	2.530100188	1.339194514	0.554823009
t00007907_to_t00022420	0.018350393	2.513332155	1.329601347	0.554823009

t00015616_to_t00015882	0.013337217	2.478504142	1.30946967	0.554823009
t00006199_to_t00029490	0.038308108	2.454527728	1.295445465	0.568363584
t00012442_to_t00013048	0.01476212	2.424873148	1.277909278	0.554823009
t00007907_to_t00015863	0.034969116	2.414222906	1.271558887	0.568363584
t00009731_to_t00013099	0.032088334	2.413820147	1.271318185	0.568363584
t00012442_to_t00016527	0.021250468	2.35859314	1.237926573	0.56259193
t00009731_to_t00015863	0.023079459	2.332031564	1.221587316	0.568363584
t00009731_to_t00021234	0.032211728	2.330558061	1.220675455	0.568363584
t00012442_to_t00015882	0.04762322	2.280469233	1.189330706	0.568363584
t00007970_to_t00029490	0.034201651	2.260239412	1.176475596	0.568363584
t00004817_to_t00028073	0.003208285	2.252945672	1.171812525	0.352911303
t00012442_to_t00020112	0.01617914	2.244294356	1.166261909	0.554823009
t00009731_to_t00014455	0.036253069	2.233770495	1.159480966	0.568363584
t00007907_to_t00021234	0.02886618	2.22168039	1.151651286	0.568363584
t00021504_to_t00028073	0.049252053	2.187972123	1.129594357	0.568363584
t00006199_to_t00027162	0.028928261	2.172556654	1.119393799	0.568363584
t00012442_to_t00015863	0.013675039	2.166705604	1.115503144	0.554823009
t00012442_to_t00021234	0.004362085	2.164437459	1.113992115	0.395153564
t00009731_to_t00010399	0.036212679	2.101179252	1.071199244	0.568363584
t00011350_to_t00016527	0.040283003	2.057109918	1.040618884	0.568363584
t00009731_to_t00013048	0.028673117	2.056369589	1.040099582	0.568363584
t00011195_to_t00020738	0.034348311	0.42104834	-1.247942218	0.568363584
t00007907_to_t00010700	0.048230368	0.409151236	-1.289293885	0.568363584
t00005497_to_t00012442	0.036389062	0.382600402	-1.386089704	0.568363584
t00008082_to_t00010700	0.045820613	0.379414042	-1.398155022	0.568363584
t00006887_to_t00020738	0.04264853	0.345062274	-1.535071346	0.568363584
t00006887_to_t00010700	0.024884722	0.319417552	-1.646484503	0.568363584
t00004817_to_t00017015	0.034257163	0.30149757	-1.729781719	0.568363584

t00006414_to_t00007970	0.046099054	0.297098533	-1.750986613	0.568363584
t00006887_to_t00017015	0.027932165	0.295121245	-1.760620316	0.568363584
t00007033_to_t00011350	0.029057313	0.287019891	-1.800777374	0.568363584
t00015882_to_t00020738	0.025257214	0.283758745	-1.817263241	0.568363584
t00014455_to_t00017015	0.045044749	0.273434472	-1.87073296	0.568363584
t00010399_to_t00010700	0.000488179	0.269171504	-1.893402411	0.173549732
t00013048_to_t00017015	0.029327023	0.267957076	-1.899926182	0.568363584
t00013099_to_t00017015	0.029910501	0.253580349	-1.979485147	0.568363584
t00020112_to_t00020738	0.020599476	0.236560214	-2.079720642	0.56259193
t00006414_to_t00010700	0.013884806	0.234881266	-2.089996446	0.554823009
t00015295_to_t00017015	6.43E-05	0.232915385	-2.102122157	0.099071121
t00009667_to_t00017015	0.049029024	0.224460791	-2.155464641	0.568363584
t00003437_to_t00020738	0.048565667	0.221374077	-2.1754418	0.568363584
t00020494_to_t00020738	0.018241622	0.21480426	-2.21890549	0.554823009
t00006414_to_t00020738	0.042811243	0.210212813	-2.25007749	0.568363584
t00016527_to_t00020738	0.019094558	0.208694053	-2.260538603	0.554823009
t00014138_to_t00017015	0.044182221	0.205611302	-2.282008523	0.568363584
t00015295_to_t00020738	0.047917969	0.162083294	-2.625192691	0.568363584
